# Crystal structure of di­aqua­(3,14-diethyl-2,6,13,17-tetra­aza­tri­cyclo­[16.4.0.0^7,12^]docosa­ne)copper(II) dichloride tetra­hydrate

**DOI:** 10.1107/S2056989021004382

**Published:** 2021-04-30

**Authors:** Dohyun Moon, Jong-Ha Choi

**Affiliations:** a Beamline Department, Pohang Accelerator Laboratory, POSTECH, Pohang 37673, Republic of Korea; b Department of Chemistry, Andong National University, Andong 36729, Republic of Korea

**Keywords:** crystal structure, macrocycle, di­aqua­copper(II) complex, hydrogen bonding, synchrotron radiation

## Abstract

The crystal structure of the novel hydrated Cu^II^ salt, [Cu(*L*)(H_2_O)_2_]Cl_2_·4H_2_O (*L* = 3,14-diethyl-2,6,13,17-tetra­aza­tri­cyclo­[16.4.0.0^7,12^]docosane, C_22_H_44_N_4_) has been determined using synchrotron radiation. The asymmetric unit contains one half of the [Cu(*L*)(H_2_O)_2_]^2+^ cation (completed by crystallographic inversion symmetry), one chloride anion and two lattice water mol­ecules. The copper(II) atom exists in a tetra­gonally distorted octa­hedral environment with the four N atoms of the macrocyclic ligand in equatorial and two O atoms from water mol­ecules in axial positions. The latter exhibit a long axial Cu—O bond length of 2.7866 (16) Å due to the Jahn–Teller distortion. The macrocyclic ring adopts a stable *trans*-III conformation with typical Cu—N bond lengths of 2.0240 (11) and 2.0441 (3) Å. The complex is stabilized by hydrogen bonds formed between the O atoms of coordinated water mol­ecules and the NH groups as donors, and chloride anions as acceptors. The chloride anions are further connected to the lattice water solvent molecules through O—H⋯Cl hydrogen bonds, giving rise to a three-dimensional network structure.

## Abstract 2

In the title complex, [Cu(C_22_H_44_N_4_)(H_2_O)_2_]Cl_2_·4H_2_O, the complex cation lies about an inversion center. The macrocyclic ring adopts a stable *trans*-III conformation. In the crystal, O—H⋯Cl, N—H⋯Cl and O—H⋯O hydrogen bonds connect the chloride anions, complex cation and water mol­ecules, forming a three-dimensional network.

## Chemical context   

The macrocycle 3,14-diethyl-2,6,13,17-tetra­aza­tri­cyclo(16.4.0.07,12)docosane (C_22_H_44_N_4_, *L*) contains a cyclam backbone with two cyclo­hexane subunits and two ethyl groups attached to carbon atoms of the propyl chains that bridge opposite pairs of N atoms. The syntheses, crystal structures and spectroscopic properties of numerous metal complexes with this ligand have previously been reported, *viz*. [Ni(*L*)(NO_3_)_2_] (Subhan & Choi, 2014 ▸), [Ni(*L*)(N_3_)_2_] (Lim *et al.*, 2015 ▸), [Ni(*L*)(NCS)_2_] (Lim & Choi, 2017 ▸), [Cu(*L*)(ClO_4_)_2_] (Lim *et al.*, 2006 ▸), [Cu(*L*)(NO_3_)_2_] and [Cu(*L*)(H_2_O)_2_](SCN)_2_ (Choi *et al.*, 2012 ▸). In these complexes, Cu^II^ or Ni^II^ cations have a tetra­gonally distorted octa­hedral coordination environment with the four N atoms of the macrocyclic ligand in the equatorial position and O/N atoms of anions or water mol­ecules in the axial position. In contrast, [Ni(*L*)](ClO_4_)_2_·2H_2_O (Subhan & Choi, 2014 ▸) and [Ni_*x*_(H_2(1–*x*)_ *L*)]Cl_2_·2H_2_O (*x* = 0.34) (Moon *et al.*, 2020 ▸) have a square-planar coordination environment around each Ni^II^ ion that binds to the four nitro­gen atoms of the macrocyclic ligand. The macrocyclic ligands in these Cu^II^ and Ni^II^ complexes adopt the most stable *trans*-III conformation. The crystal structures of (*L*)·NaClO_4_ (Aree *et al.*, 2018 ▸), [H_2_ *L*](ClO_4_)_2_ (Aree *et al.*, 2018 ▸), [H_2_ *L*]Cl_2_·4H_2_O (Moon *et al.*, 2013 ▸), [H_2_ *L*](NO_3_)_2_·2H_2_O (Moon *et al.*, 2019 ▸) and [H_4_ *L*]Cl_4_·4H_2_O (Moon & Choi, 2021 ▸) have also been determined.
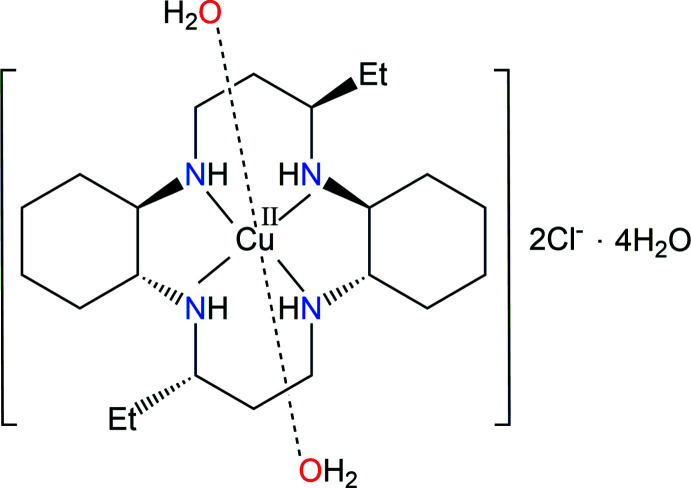


We report here synthesis and structural characterization of the novel complex [Cu(*L*)(H_2_O)_2_]Cl_2_·4H_2_O, (I), in order to obtain detailed information on the conformation of the macrocyclic ligand, and the bonding mode of water mol­ecules and chloride anions in the crystal.

## Structural commentary   

**Figure 1:**
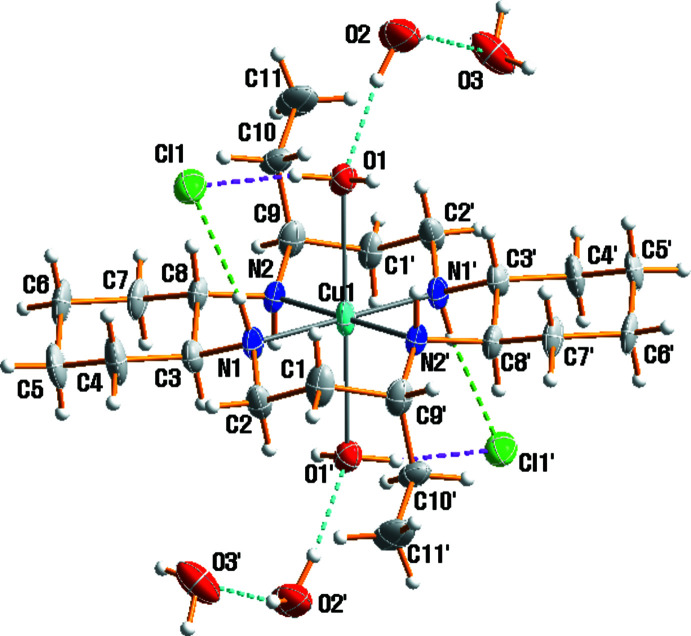
Mol­ecular structure of (I), drawn with displacement ellipsoids at the 50% probability level. Dashed lines represent hydrogen-bonding inter­actions; primed atoms are related by the symmetry operation (−*x* + 1, −*y* + 1, −*z* + 1).

The mol­ecular structure of (I) is shown in Fig. 1 ▸. The Cu^II^ complex cation lies across a crystallographic inversion center, and hence the asymmetric unit consists of one half of the [Cu(*L*)(H_2_O)_2_]^2+^ cation, one chloride anion and two lattice water solvents. The macrocyclic skeleton adopts the most stable *trans*-III conformation. The Cu—N bond lengths [2.0240 (11)–2.0441 (3) Å] are within the typical range, and are comparable to those observed in related complexes, *e.g.* in [Cu(*L*)(ClO_4_)_2_] [2.01064 (18)–2.0403 (18) Å] (Lim *et al.*, 2006 ▸), [Cu(*L*)(NO_3_)_2_] [2.021 (2)–2.046 (2) Å] and [Cu(*L*)(H_2_O)_2_](SCN)_2_ [2.014 (2)–2.047 (2) Å] (Choi *et al.*, 2012 ▸). The coordination environment of the copper(II) atom may be considered as square-planar or octa­hedral with a tetra­gonal distortion, depending upon whether or not the remote oxygen atoms of the water mol­ecules are considered to be bonded to the copper(II) atom. The concept of a semi-coordinating atom was introduced to describe a situation where a polyatomic anion or ligand occupies the long axial position in an otherwise square-planar copper(II) complex with an atom in the distance range of 2.5–3.0 Å (Murphy & Hathaway, 2003 ▸). The axial Cu1—O1 distance of 2.7866 (16) Å is longer than corresponding distances in [Cu(*L*)(ClO_4_)_2_] [2.762 (2) Å] (Lim *et al.*, 2006 ▸), [Cu(*L*)(NO_3_)_2_] [2.506 (2) Å] and [Cu(*L*)(H_2_O)_2_](SCN)_2_ [2.569 (2) Å] (Choi *et al.*, 2012 ▸). The tetra­gonally elongated octa­hedron is a common polyhedron around six-coordinate Cu^II^ atoms in complexes (involving also non-equivalent ligands), and the distortion arises from the Jahn–Teller effect operative on the metal cation with its *d* ^9^ electronic configuration (Murphy & Hathaway, 2003 ▸).

The two ethyl groups on the six-membered chelate rings and the two –(CH_2_)_4_– parts of the cyclo­hexane backbones are *anti* with respect to the macrocyclic plane. As usually observed, the five-membered chelate rings adopt a *gauche* conformation whereas the six-membered rings are in chair conformations. The ethyl groups are attached axially as substituents to the six-membered rings, while the methyl­ene C substituents at the five-membered rings are equatorial. The cyclo­hexane rings are also in a chair conformation, with the N substituents in equatorial positions.

## Supra­molecular features   

**Figure 2:**
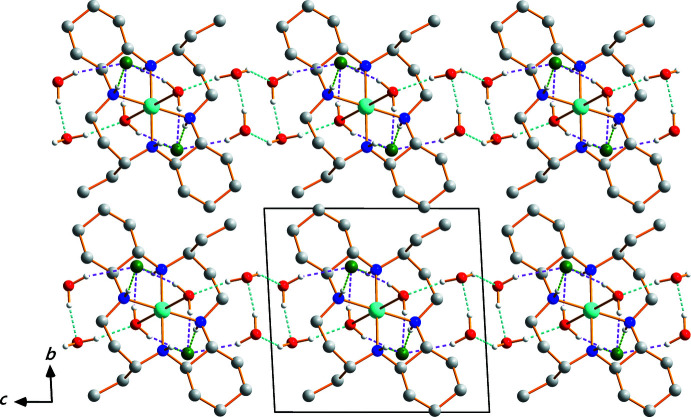
Crystal packing of (I), viewed perpendicular to the *bc* plane. Dashed lines represent O—H⋯Cl (purple), O—H⋯O (cyan), and N—H⋯Cl (green) hydrogen-bonding inter­actions, respectively. H atoms bound to C atoms have been omitted for clarity.

Numerical details of the hydrogen bonding are given in Table 1 ▸. The supra­molecular structure involves inter­actions between the NH groups of the macrocycle and OH groups of the semi-coordinated water mol­ecules as donors, and the chloride anions and the O atoms of the lattice water mol­ecules as acceptors, resulting in a three-dimensional network structure. The chloride anions remain outside the coordination sphere [Cu⋯Cl (4.523 Å)] and are connected both to the semi-coordinated and to the lattice water solvents through O—H⋯Cl hydrogen bonds. The lattice water solvents are additionally linked to the semi-coordinated water mol­ecules and other lattice water solvents *via* O—H⋯O hydrogen bonds. The crystal packing of (I) in a view perpendicular to the *bc* plane is shown in Fig. 2 ▸.

## Database survey   

A search of the Cambridge Structural (Version 5.42, update February 2021; Groom *et al.*, 2016 ▸) indicated 21 hits for organic and transition-metal compounds containing the macrocycle (*L*, C_22_H_44_N_4_). The hits include (*L*)·NaClO_4_ (Aree *et al.*, 2018 ▸), [H_2_ *L*](ClO_4_)_2_ (Aree *et al.*, 2018 ▸), [H_2_ *L*]Cl_2_·4H_2_O (Moon *et al.*, 2013 ▸), [H_2_ *L*](NO_3_)_2_·2H_2_O (Moon *et al.*, 2019 ▸), [H_4_ *L*]Cl_4_·4H_2_O (Moon & Choi, 2021 ▸), [Cu(*L*)(ClO_4_)_2_] (Lim *et al.*, 2006 ▸), [Cu(*L*)(NO_3_)_2_] and [Cu(*L*)(H_2_O)_2_](SCN)_2_ (Choi *et al.*, 2012 ▸). Until now, no crystal structure of the [Cu(*L*)(H_2_O)_2_]^2+^ cation with chloride counter-anions and four lattice water mol­ecules has been deposited.

## Synthesis and crystallization   

Ethyl vinyl ketone (97%), *trans*-1,2-cyclo­hexa­nedi­amine (99%) and copper(II) chloride dihydrate (99%) were purchased from Sigma-Aldrich and were used as received. All other chemicals were analytical reagent grade. 3,14-Diethyl-2,6,13,17-tetra­aza­tri­cyclo­(16.4.0.0^7,12^)docosane (*L*) was prepared according to a published procedure (Lim *et al.*, 2006 ▸). A solution of the macrocycle *L* (0.091 g, 0.25 mmol) in water (10 mL) was added dropwise to a stirred solution of CuCl_2_·2H_2_O (0.085 g, 0.5 mmol) in water (20 mL). After cooling to 298 K, the pH was adjusted to 3.0 by the addition of 1.0 *M* HCl. A mixture of colorless and violet crystals had formed from the solution over the next few days. To the mixture were added 30 mL of MeOH under stirring, and the stirring was continued for 30 min. The colourless crystals of [H_4_ *L*]Cl_4_·4H_2_O (Moon & Choi, 2021 ▸) were removed by filtration. The filtrate was left at 298 K. After few days, plate-like violet single crystals of (I) suitable for X-ray analysis were obtained.

## Refinement   

Crystal data, data collection and structure refinement details are summarized in Table 2 ▸. All C- and N-bound H atoms in the complex were placed in geometrically idealized positions and constrained to ride on their parent atoms, with C—H distances of 0.97–0.99 Å, and with an N—H distance of 0.99 Å with *U*_iso_(H) values of 1.2 and 1.5 *U*_eq_ of the parent atoms, respectively. The hydrogen atoms of the water mol­ecules were found in difference-Fourier maps, and were restrained using DFIX and DANG commands during the least-squares refinement with *U*_iso_(H) values of 1.2*U*_eq_ of the oxygen atom.

## Supplementary Material

Crystal structure: contains datablock(s) I. DOI: 10.1107/S2056989021004382/wm5607sup1.cif

Structure factors: contains datablock(s) I. DOI: 10.1107/S2056989021004382/wm5607Isup2.hkl

CCDC reference: 2079818

Additional supporting information:  crystallographic information; 3D view; checkCIF report

## Figures and Tables

**Table 1:** Hydrogen-bond geometry (Å, °)

*D*—H⋯*A*	*D*—H	H⋯*A*	*D*⋯*A*	*D*—H⋯*A*
N1—H1⋯Cl1	0.99	2.45	3.4383 (14)	173
N2—H2⋯Cl1^i^	0.99	2.54	3.4962 (14)	163
O1—H1*O*1⋯Cl1^ii^	0.92 (1)	2.26 (1)	3.1799 (19)	173 (2)
O1—H2*O*1⋯Cl1	0.93 (1)	2.21 (1)	3.1153 (15)	166 (2)
O2—H1*O*2⋯O1	0.93 (1)	1.98 (1)	2.902 (2)	172 (3)
O2—H2*O*2⋯O3	0.93 (1)	1.94 (2)	2.794 (3)	152 (3)
O3—H1*O*3⋯Cl1^iii^	0.93 (1)	2.39 (2)	3.266 (2)	157 (3)
O3—H2*O*3⋯O2^iv^	0.93 (1)	1.87 (1)	2.796 (4)	170 (3)

Symmetry codes: (i) x-1, y, z; (ii) -x+2, -y+1, -z+1; (iii) x, y, z-1; (iv) -x+2, -y+1, -z.

**Table 2:** Experimental details

Crystal data	
Chemical formula	[Cu(C_22_H_44_N_4_)(H_2_O)_2_]Cl_2_·4H_2_O
*M*_r_	607.14
Crystal system, space group	Triclinic, *P*\overline{1}
Temperature (K)	220
*a*, *b*, *c* (Å)	8.0220 (16), 10.020 (2), 10.354 (2)
α, β, γ (°)	81.36 (3), 72.84 (3), 69.71 (3)
*V* (Å^3^)	744.8 (3)
*Z*	1
Radiation type	Synchrotron, λ = 0.610 Å
μ (mm^−1^)	0.63
Crystal size (mm)	0.12 × 0.12 × 0.04

Data collection	
Diffractometer	Rayonix MX225HS CCD area detector
Absorption correction	Empirical (using intensity measurements) (*HKL3000sm *SCALEPACK**; Otwinowski *et al.*, 2003 ▸)
*T*_min_, *T*_max_	0.597, 1.000
No. of measured, independent and observed [*I* > 2σ(*I*)] reflections	8262, 4141, 4013
*R*_int_	0.019
(sin θ/λ)_max_ (Å^−1^)	0.693

Refinement	
*R*[*F* ^2^ > 2σ(*F* ^2^)], *wR*(*F* ^2^), *S*	0.039, 0.109, 1.10
No. of reflections	4141
No. of parameters	179
No. of restraints	9
H-atom treatment	H atoms treated by a mixture of independent and constrained refinement
Δρ_max_, Δρ_min_ (e Å^−3^)	0.86, −0.88

Computer programs: *PAL BL2D-SMDC* (Shin *et al.*, 2016 ▸), *HKL3000sm* (Otwinowski *et al.*, 2003 ▸), *SHELXT2018* (Sheldrick, 2015*a* ▸), *SHELXL2018* (Sheldrick, 2015*b* ▸), *DIAMOND* (Putz & Brandenburg, 2014 ▸) and *publCIF* (Westrip, 2010 ▸).

## supplementary crystallographic information

### Crystal data

**Table:**

[Cu(C_22_H_44_N_4_)(H_2_O)_2_]Cl_2_·4H_2_O	*Z* = 1
*M**_r_* = 607.14	*F*(000) = 327
Triclinic, *P*1	*D*_x_ = 1.354 Mg m^−^^3^
*a* = 8.0220 (16) Å	Synchrotron radiation, λ = 0.610 Å
*b* = 10.020 (2) Å	Cell parameters from 49370 reflections
*c* = 10.354 (2) Å	θ = 0.4–33.7°
α = 81.36 (3)°	µ = 0.63 mm^−^^1^
β = 72.84 (3)°	*T* = 220 K
γ = 69.71 (3)°	Plate, violet
*V* = 744.8 (3) Å^3^	0.12 × 0.12 × 0.04 mm

### Data collection

**Table:**

Rayonix MX225HS CCD area detector diffractometer	4013 reflections with *I* > 2σ(*I*)
Radiation source: PLSII 2D bending magnet	*R*_int_ = 0.019
ω scan	θ_max_ = 25.0°, θ_min_ = 1.8°
Absorption correction: empirical (using intensity measurements) (*HKL3000sm Scalepack*; Otwinowski *et al.*, 2003)	*h* = −11→11
*T*_min_ = 0.597, *T*_max_ = 1.000	*k* = −13→13
8262 measured reflections	*l* = −14→14
4141 independent reflections	

### Refinement

**Table:**

Refinement on *F*^2^	9 restraints
Least-squares matrix: full	Hydrogen site location: mixed
*R*[*F*^2^ > 2σ(*F*^2^)] = 0.039	H atoms treated by a mixture of independent and constrained refinement
*wR*(*F*^2^) = 0.109	*w* = 1/[σ^2^(*F*_o_^2^) + (0.074*P*)^2^ + 0.2905*P*] where *P* = (*F*_o_^2^ + 2*F*_c_^2^)/3
*S* = 1.10	(Δ/σ)_max_ < 0.001
4141 reflections	Δρ_max_ = 0.86 e Å^−^^3^
179 parameters	Δρ_min_ = −0.88 e Å^−^^3^

### Special details

**Table:**

Geometry. All esds (except the esd in the dihedral angle between two l.s. planes) are estimated using the full covariance matrix. The cell esds are taken into account individually in the estimation of esds in distances, angles and torsion angles; correlations between esds in cell parameters are only used when they are defined by crystal symmetry. An approximate (isotropic) treatment of cell esds is used for estimating esds involving l.s. planes.

### Fractional atomic coordinates and isotropic or equivalent isotropic displacement parameters (Å^2^)

**Table:**

	*x*	*y*	*z*	*U*_iso_*/*U*_eq_	
Cu1	0.500000	0.500000	0.500000	0.01142 (9)	
N1	0.51043 (14)	0.55835 (11)	0.67558 (11)	0.01005 (19)	
H1	0.615585	0.595592	0.652199	0.012*	
N2	0.31184 (14)	0.69854 (11)	0.49340 (11)	0.00997 (19)	
H2	0.191904	0.681599	0.531917	0.012*	
C1	0.71839 (18)	0.32162 (13)	0.73164 (14)	0.0148 (2)	
H1A	0.755423	0.264611	0.810137	0.018*	
H1B	0.816429	0.361734	0.682545	0.018*	
C2	0.5438 (2)	0.44417 (14)	0.78296 (13)	0.0167 (2)	
H2A	0.438576	0.408346	0.814450	0.020*	
H2B	0.554800	0.483575	0.860014	0.020*	
C3	0.34162 (16)	0.68194 (12)	0.72187 (12)	0.0110 (2)	
H3	0.233945	0.647336	0.753181	0.013*	
C4	0.3491 (2)	0.75727 (15)	0.83728 (14)	0.0188 (3)	
H4A	0.352331	0.692132	0.917871	0.023*	
H4B	0.461760	0.783661	0.810983	0.023*	
C5	0.1815 (2)	0.89088 (16)	0.87053 (16)	0.0224 (3)	
H5A	0.069628	0.863517	0.904981	0.027*	
H5B	0.191875	0.939817	0.941531	0.027*	
C6	0.1665 (2)	0.99167 (15)	0.74592 (16)	0.0209 (3)	
H6A	0.273938	1.024974	0.715722	0.025*	
H6B	0.056173	1.074922	0.769256	0.025*	
C7	0.15596 (18)	0.91777 (14)	0.63083 (15)	0.0166 (3)	
H7A	0.042053	0.893014	0.657577	0.020*	
H7B	0.153337	0.983052	0.550302	0.020*	
C8	0.32144 (16)	0.78275 (12)	0.59713 (12)	0.0103 (2)	
H8	0.434051	0.810898	0.561857	0.012*	
C9	0.29426 (17)	0.77801 (12)	0.36087 (13)	0.0123 (2)	
H9	0.175786	0.856733	0.379043	0.015*	
C10	0.4461 (2)	0.84486 (16)	0.29985 (15)	0.0206 (3)	
H10A	0.565865	0.770339	0.290927	0.025*	
H10B	0.436977	0.913687	0.361642	0.025*	
C11	0.4366 (3)	0.9202 (2)	0.16157 (18)	0.0339 (4)	
H11A	0.471172	0.849892	0.095303	0.051*	
H11B	0.520561	0.975525	0.135068	0.051*	
H11C	0.312058	0.982978	0.166352	0.051*	
Cl1	0.85009 (5)	0.71364 (5)	0.60787 (4)	0.02792 (11)	
O1	0.81875 (19)	0.57072 (14)	0.37263 (14)	0.0307 (3)	
H1O1	0.919 (2)	0.4892 (16)	0.370 (3)	0.037*	
H2O1	0.823 (3)	0.628 (2)	0.433 (2)	0.037*	
O2	0.9753 (3)	0.6599 (2)	0.09693 (19)	0.0573 (5)	
H1O2	0.919 (4)	0.641 (4)	0.1865 (13)	0.069*	
H2O2	0.897 (4)	0.678 (4)	0.041 (2)	0.069*	
O3	0.7816 (3)	0.6189 (3)	−0.06985 (19)	0.0642 (6)	
H1O3	0.813 (5)	0.665 (3)	−0.154 (2)	0.077*	
H2O3	0.855 (4)	0.5235 (15)	−0.068 (3)	0.077*	

### Atomic displacement parameters (Å^2^)

**Table:**

	*U*^11^	*U*^22^	*U*^33^	*U*^12^	*U*^13^	*U*^23^
Cu1	0.01753 (13)	0.00598 (12)	0.00936 (12)	0.00037 (8)	−0.00540 (8)	−0.00249 (7)
N1	0.0121 (4)	0.0065 (4)	0.0101 (4)	0.0000 (3)	−0.0032 (3)	−0.0025 (3)
N2	0.0125 (4)	0.0069 (4)	0.0112 (5)	−0.0020 (3)	−0.0045 (4)	−0.0025 (3)
C1	0.0184 (6)	0.0116 (5)	0.0161 (6)	−0.0007 (4)	−0.0110 (5)	−0.0022 (4)
C2	0.0239 (6)	0.0112 (5)	0.0110 (5)	0.0002 (5)	−0.0055 (5)	−0.0009 (4)
C3	0.0113 (5)	0.0086 (5)	0.0113 (5)	−0.0002 (4)	−0.0021 (4)	−0.0038 (4)
C4	0.0223 (6)	0.0163 (6)	0.0152 (6)	0.0024 (5)	−0.0066 (5)	−0.0101 (5)
C5	0.0214 (6)	0.0199 (6)	0.0207 (7)	0.0029 (5)	−0.0029 (5)	−0.0139 (5)
C6	0.0216 (6)	0.0119 (6)	0.0291 (7)	0.0025 (5)	−0.0105 (5)	−0.0120 (5)
C7	0.0156 (6)	0.0098 (5)	0.0232 (6)	0.0033 (4)	−0.0087 (5)	−0.0079 (5)
C8	0.0114 (5)	0.0068 (5)	0.0128 (5)	−0.0005 (4)	−0.0043 (4)	−0.0038 (4)
C9	0.0155 (5)	0.0078 (5)	0.0135 (5)	−0.0003 (4)	−0.0077 (4)	−0.0004 (4)
C10	0.0295 (7)	0.0207 (6)	0.0173 (6)	−0.0149 (6)	−0.0095 (5)	0.0054 (5)
C11	0.0573 (11)	0.0326 (9)	0.0209 (7)	−0.0262 (8)	−0.0163 (7)	0.0126 (6)
Cl1	0.02403 (19)	0.0315 (2)	0.0321 (2)	−0.01328 (15)	−0.00442 (15)	−0.00825 (16)
O1	0.0371 (7)	0.0302 (6)	0.0299 (6)	−0.0124 (5)	−0.0141 (5)	−0.0017 (5)
O2	0.0797 (13)	0.0716 (12)	0.0341 (8)	−0.0444 (11)	−0.0144 (8)	0.0048 (8)
O3	0.0475 (10)	0.1017 (17)	0.0319 (8)	−0.0083 (10)	−0.0118 (7)	−0.0040 (9)

### Geometric parameters (Å, º)

**Table:**

Cu1—N1^i^	2.0240 (11)	C5—C6	1.521 (2)
Cu1—N1	2.0240 (11)	C5—H5A	0.9800
Cu1—N2	2.0441 (13)	C5—H5B	0.9800
Cu1—N2^i^	2.0441 (13)	C6—C7	1.5310 (19)
Cu1—O1^i^	2.7866 (16)	C6—H6A	0.9800
Cu1—O1	2.7866 (16)	C6—H6B	0.9800
N1—C2	1.4826 (17)	C7—C8	1.5288 (18)
N1—C3	1.4932 (16)	C7—H7A	0.9800
N1—H1	0.9900	C7—H7B	0.9800
N2—C8	1.4958 (15)	C8—H8	0.9900
N2—C9	1.4983 (16)	C9—C10	1.5216 (19)
N2—H2	0.9900	C9—H9	0.9900
C1—C2	1.5217 (19)	C10—C11	1.525 (2)
C1—C9^i^	1.5295 (17)	C10—H10A	0.9800
C1—H1A	0.9800	C10—H10B	0.9800
C1—H1B	0.9800	C11—H11A	0.9700
C2—H2A	0.9800	C11—H11B	0.9700
C2—H2B	0.9800	C11—H11C	0.9700
C3—C8	1.5272 (18)	O1—H1O1	0.924 (9)
C3—C4	1.5315 (18)	O1—H2O1	0.928 (9)
C3—H3	0.9900	O2—H1O2	0.927 (10)
C4—C5	1.528 (2)	O2—H2O2	0.931 (10)
C4—H4A	0.9800	O3—H1O3	0.931 (10)
C4—H4B	0.9800	O3—H2O3	0.934 (10)
			
N1^i^—Cu1—N1	180.0	C3—C4—H4B	109.5
N1^i^—Cu1—N2	95.42 (5)	H4A—C4—H4B	108.1
N1—Cu1—N2	84.58 (5)	C6—C5—C4	111.05 (12)
N1^i^—Cu1—N2^i^	84.58 (5)	C6—C5—H5A	109.4
N1—Cu1—N2^i^	95.42 (5)	C4—C5—H5A	109.4
N2—Cu1—N2^i^	180.0	C6—C5—H5B	109.4
N1^i^—Cu1—O1^i^	88.11 (5)	C4—C5—H5B	109.4
N1—Cu1—O1^i^	91.89 (5)	H5A—C5—H5B	108.0
N2—Cu1—O1^i^	81.60 (5)	C5—C6—C7	111.09 (12)
N2^i^—Cu1—O1^i^	98.40 (5)	C5—C6—H6A	109.4
N1^i^—Cu1—O1	91.89 (5)	C7—C6—H6A	109.4
N1—Cu1—O1	88.11 (5)	C5—C6—H6B	109.4
N2—Cu1—O1	98.40 (5)	C7—C6—H6B	109.4
N2^i^—Cu1—O1	81.60 (5)	H6A—C6—H6B	108.0
O1^i^—Cu1—O1	180.00 (5)	C8—C7—C6	110.69 (11)
C2—N1—C3	113.32 (10)	C8—C7—H7A	109.5
C2—N1—Cu1	116.78 (8)	C6—C7—H7A	109.5
C3—N1—Cu1	107.51 (8)	C8—C7—H7B	109.5
C2—N1—H1	106.2	C6—C7—H7B	109.5
C3—N1—H1	106.2	H7A—C7—H7B	108.1
Cu1—N1—H1	106.2	N2—C8—C3	106.51 (9)
C8—N2—C9	115.20 (9)	N2—C8—C7	113.49 (10)
C8—N2—Cu1	107.77 (8)	C3—C8—C7	111.87 (11)
C9—N2—Cu1	120.89 (8)	N2—C8—H8	108.3
C8—N2—H2	103.6	C3—C8—H8	108.3
C9—N2—H2	103.6	C7—C8—H8	108.3
Cu1—N2—H2	103.6	N2—C9—C10	111.93 (10)
C2—C1—C9^i^	116.20 (11)	N2—C9—C1^i^	108.49 (10)
C2—C1—H1A	108.2	C10—C9—C1^i^	114.34 (11)
C9^i^—C1—H1A	108.2	N2—C9—H9	107.2
C2—C1—H1B	108.2	C10—C9—H9	107.2
C9^i^—C1—H1B	108.2	C1^i^—C9—H9	107.2
H1A—C1—H1B	107.4	C9—C10—C11	112.89 (13)
N1—C2—C1	111.40 (11)	C9—C10—H10A	109.0
N1—C2—H2A	109.3	C11—C10—H10A	109.0
C1—C2—H2A	109.3	C9—C10—H10B	109.0
N1—C2—H2B	109.3	C11—C10—H10B	109.0
C1—C2—H2B	109.3	H10A—C10—H10B	107.8
H2A—C2—H2B	108.0	C10—C11—H11A	109.5
N1—C3—C8	106.11 (10)	C10—C11—H11B	109.5
N1—C3—C4	113.33 (10)	H11A—C11—H11B	109.5
C8—C3—C4	111.49 (10)	C10—C11—H11C	109.5
N1—C3—H3	108.6	H11A—C11—H11C	109.5
C8—C3—H3	108.6	H11B—C11—H11C	109.5
C4—C3—H3	108.6	Cu1—O1—H1O1	108.4 (16)
C5—C4—C3	110.76 (12)	Cu1—O1—H2O1	103.4 (16)
C5—C4—H4A	109.5	H1O1—O1—H2O1	106.2 (16)
C3—C4—H4A	109.5	H1O2—O2—H2O2	113 (2)
C5—C4—H4B	109.5	H1O3—O3—H2O3	111 (2)
			
C3—N1—C2—C1	−179.24 (10)	C9—N2—C8—C7	57.28 (14)
Cu1—N1—C2—C1	54.98 (13)	Cu1—N2—C8—C7	−164.43 (9)
C9^i^—C1—C2—N1	−75.35 (15)	N1—C3—C8—N2	57.32 (12)
C2—N1—C3—C8	−175.98 (10)	C4—C3—C8—N2	−178.85 (10)
Cu1—N1—C3—C8	−45.40 (10)	N1—C3—C8—C7	−178.14 (10)
C2—N1—C3—C4	61.34 (14)	C4—C3—C8—C7	−54.31 (14)
Cu1—N1—C3—C4	−168.07 (9)	C6—C7—C8—N2	175.13 (11)
N1—C3—C4—C5	174.44 (11)	C6—C7—C8—C3	54.58 (15)
C8—C3—C4—C5	54.79 (16)	C8—N2—C9—C10	54.25 (14)
C3—C4—C5—C6	−56.45 (16)	Cu1—N2—C9—C10	−78.16 (12)
C4—C5—C6—C7	57.37 (16)	C8—N2—C9—C1^i^	−178.65 (10)
C5—C6—C7—C8	−56.05 (16)	Cu1—N2—C9—C1^i^	48.93 (12)
C9—N2—C8—C3	−179.19 (9)	N2—C9—C10—C11	177.17 (13)
Cu1—N2—C8—C3	−40.90 (10)	C1^i^—C9—C10—C11	53.30 (17)

Symmetry code: (i) −*x*+1, −*y*+1, −*z*+1.

### Hydrogen-bond geometry (Å, º)

**Table:**

*D*—H···*A*	*D*—H	H···*A*	*D*···*A*	*D*—H···*A*
N1—H1···Cl1	0.99	2.45	3.4383 (14)	173
N2—H2···Cl1^ii^	0.99	2.54	3.4962 (14)	163
O1—H1*O*1···Cl1^iii^	0.92 (1)	2.26 (1)	3.1799 (19)	173 (2)
O1—H2*O*1···Cl1	0.93 (1)	2.21 (1)	3.1153 (15)	166 (2)
O2—H1*O*2···O1	0.93 (1)	1.98 (1)	2.902 (2)	172 (3)
O2—H2*O*2···O3	0.93 (1)	1.94 (2)	2.794 (3)	152 (3)
O3—H1*O*3···Cl1^iv^	0.93 (1)	2.39 (2)	3.266 (2)	157 (3)
O3—H2*O*3···O2^v^	0.93 (1)	1.87 (1)	2.796 (4)	170 (3)

Symmetry codes: (ii) *x*−1, *y*, *z*; (iii) −*x*+2, −*y*+1, −*z*+1; (iv) *x*, *y*, *z*−1; (v) −*x*+2, −*y*+1, −*z*.
